# The anti-politics of food in South Africa: transformation, accountability and the nutrition policy subsystem

**DOI:** 10.1017/S1368980025000163

**Published:** 2025-02-03

**Authors:** Busiso Helard Moyo, Anne Marie Thow, Florian Kroll, Scott Drimie

**Affiliations:** 1 Faculty of Community and Health Sciences, School of Public Health & DST-NRF Centre of Excellence in Food Security (CoE-FS), University of the Western Cape, Robert Sobukwe Rd, Bellville, Cape Town 7535, South Africa; 2 School of Public Health, The University of Sydney, Camperdown 2006, NSW, Australia; 3 Institute for Poverty, Land and Agrarian Studies (PLAAS), University of the Western Cape, Private Bag X17, Bellville 7535, South Africa; 4 Department of Global Health, Faculty of Health and Medicine Sciences, University of Stellenbosch, Private Bag X1, Matieland 7602, South Africa

**Keywords:** Right to food, Nutrition, South Africa, Malnutrition, Double burden, Policy, Sustainable Development Goals, Food sovereignty

## Abstract

**Objective:**

To examine power and governance arrangements in food and nutrition policy formulation and agenda setting in South Africa.

**Design:**

Analysis of the policy implementation environment and in-depth interviews were conducted focusing on: existing policy content and priorities across food system sectors; institutional structures for cross-sectoral and external stakeholder engagement; exercise of power in relation to food system policies and opportunities to strengthen action on nutrition.

**Setting:**

South Africa.

**Participants:**

Interviews were conducted with forty-eight key stakeholders involved in the food and nutrition policy sphere: government sectors relevant to food systems (*n* 21), the private sector (*n* 4), academia (*n* 10), non-government organisations (*n* 11) and farmers (*n* 2).

**Results:**

This study found that there are power dynamics involved in shaping the planning agenda that is inadvertently generating a food system that undermines the right to food. The concept of nutrition governance remains poorly defined and applied in different ways and usually based on a relatively narrow interpretation – therefore limiting policy coherence and coordination. South Africa has strong legal institutions and practices, and social policies that support public provisioning of food, but a non-interventionist approach to the food system.

**Conclusions:**

The right to food and nutrition, as outlined in the South African Constitution, has not yet been effectively utilised to establish a robust normative and legal basis for tackling the dual challenges of food insecurity and malnutrition. Currently, the governance of the food system is grappling with substantial obstacles, balancing the influence of powerful stakeholders who uphold the status quo against its responsibilities for food justice.

At present, there is overwhelming evidence that the national food system in South Africa is in crisis^(1)^. The multiple burdens of malnutrition (the co-existence of undernutrition, overnutrition and micronutrient deficiencies) and food insecurity in South Africa exist despite the availability of sufficient food at the national level. This is due to inequities in food distribution and access to food and to wider South African social inequity^(2)^. Over half of all South Africans are reported as food insecure, and nearly one in four children under the age of three have their growth stunted by malnutrition^(3)^. Taking into account the devastating effects of the COVID-19 pandemic – food insecurity rates have risen, with more than 40 % of the population classified as food insecure in 2020^(4)^. Poverty is a key contributor to poor nutrition; almost two in five South Africans do not have enough money to purchase adequate food and essential items, and according to Statistics South Africa (Stats SA) an estimated 21 % of households experienced hunger in 2017^(5)^. Current diets are also environmentally and socially unsustainable^(6)^, and the health burden induced by the country’s diet is significant. In particular, obesity and other non-communicable diseases have risen rapidly, resulting in illness, preventable deaths and high healthcare cost^(7)^. The availability and affordability of processed, high-energy, nutrient-poor foods is greater than that of fresh, healthy food”^(8)^. Notably, in 2016, the Demographic and Health Survey reported that 68 % of women were obese (BMI > 30) and/or overweight (BMI > 25), and 31 % of men were overweight or obese^(9)^. This situation is further confounded by an agrarian system that remains ‘highly dualistic – with a commercial farming sector producing most of the food, juxtaposed against a large number of smallholder and subsistence farmers that remain marginalised from the dominant system’^(10)^.

There is growing recognition that food security and nutrition are human rights issues in South Africa^(11,12)^. Under the right to food, individuals have a right to food that is available, accessible, adequate and sustainable and can seek to hold governments and other duty bearers to account for failures to protect and respect their rights^(13)^. The right to food is codified in the International Covenant on Economic, Social and Cultural Rights, this is the main international instrument and in South Africa it is enshrined in Section 27 of the Bill of Rights, which includes an imperative to address malnutrition and promote good health^(14)^. The country has a uniquely food and nutrition-friendly constitution that further supports the right to nutrition for children (in Section 28) and prisoners (Section 35(2e)). This mandate is the responsibility of the government – namely the legislature, executive and the judiciary. In other words, the South African government is obliged to progressively realise the right to food and basic nutrition, meaning it must ensure that the enjoyment of this right is consistently expanded over time, towards an end goal of a universal and full enjoyment of the right. The government of South Africa has adopted a range of instruments, policies and programmes aimed at improving national food security (Table 1 – Appendix). Overarching developmental policies of government have alluded to and addressed the imperative of fulfilling the right to food and nutrition for all. However, despite these varied initiatives and policies, the South African government is yet to operationalise the right to food in a way that delivers a nourishing food system that caters to all people^(1)^. Policy initiatives to date have proven insufficient to improve the food and nutrition security status of the population as the ‘slow violence of malnutrition’ continues^(15)^. Legislation to realise the right to food is fragmented, and there is not a focused mandate within the existing government structure^(16,17)^. Moreover, there appears to be an overemphasis on production and utilisation within the country’s food systems whilst the processing and distribution side, which is contributing to growing overweight and obesity, is being ignored^(18)^. Malnutrition in all its forms remains prevalent despite a proliferation of policies, programmes and initiatives designed to eliminate it^(15)^.

However, creating policies that address the multiple dimensions of malnutrition and food security (including social, political, economic, environmental, etc.) has proved challenging given the array of multiple actor priorities and agendas. Recent studies examining governance and policy dynamics in the South African food system have highlighted the tensions and contestations between actors around the agenda setting for different food system objectives^(15,19–21)^. These tensions exist across several lines, including economic priorities *v*. social equity, with powerful corporate actors prioritising profit-driven models of production and distribution, often at odds with the needs of marginalised communities seeking affordable and nutritious food. At the local level, smallholder farmers and informal traders often clash with large-scale commercial producers and supermarket chains, as the latter’s dominance in the food value chain marginalises smaller actors and limits opportunities for local production and distribution. There are also environmental sustainability objectives – such as promoting agroecological practices – that are frequently overshadowed by industrial agricultural models that prioritise monoculture and high-input farming, exacerbating ecological degradation and resource inequality. These competing priorities and power asymmetries create a deeply fragmented governance landscape, where aligning food system objectives remains a significant challenge. To re-design food systems for better food and nutrition security outcomes, we need to understand the in-built tendency for powerful actors in the system to control the narrative framings that shape policy design. These narratives push back on attempted changes and thus maintain the status quo^(22)^.

Analysing these dynamics through a human rights lens offers a framework for addressing the underlying social determinants that contribute to food insecurity and, more importantly, human rights are accompanied by legally binding obligations on state actors^(12,17,23)^. A human rights lens to food and nutrition governance provides a counterbalance to the dominant neoliberal food system narrative in South Africa^(23,24)^. It promotes the right to nutritious food for all and requires the equitable distribution of resources especially for marginalised and poor communities.

A food systems approach^(15,25)^ further clarifies that food cannot be dealt with appropriately when approached in the fragmented way that the current global institutional architecture of prevailing food governance encourages. Candel^(26)^ argues that the food governance system should be made more coherent and harmonised, better integrated and coordinated and more inclusive^(26)^. Responding to everyday hunger, the burden of malnutrition and the longer-term health effects of food poverty in South Africa thus call for a re-think of the political and economic forces that have created food environments that leave many eking out an existence on cheap, unhealthy foods^(12,27)^. Adopting a political lens raises questions of power and influence in the food system, enabling analysis of the influence of dominant actors on food and nutrition policy^(28)^. An analysis of food systems must therefore include power as an aspect of political economy, in order to understand how power relations have developed and in-turn affected different food system actors^(19,29)^.

This study presents an analysis of nutrition governance in light of the South African government’s commitment to fulfil the right to food and address the multiple burden of malnutrition^(30)^. The aim of this paper is to describe food system governance in South African, drawing on frameworks of human rights and power, and to analyse governance arrangements for food system transformation. This analysis addresses the urgent need to shift power relations away from dominant actors who reinforce the embedded inequities and lock-ins that keep current unsatisfactory systems in place.

## Methods

### Study design

This study used policy analysis methods to address the primary research question: *What is the governance structure and power discourse, guiding South Africa’s response to the multiple burden of malnutrition? (Including the interface with economic and social factors).* We conducted semi-structured, in-depth interviews (*n* 48) with key actors engaged-in South Africa’s food and nutrition policy sphere to obtain data on the process and people who influence food policy making in South Africa, potential opportunities for consideration of nutrition policy goals in food policy making and potential policy opportunities to address specific food commodities associated with non-communicable disease prevention or risk.

### Theoretical framing

We draw on Clapp & Fuchs^(31)^ conceptions of power for research on global food and agriculture governance, which lays out instrumental, discursive and structural power, as an adaptation of Lukes’ (2005) ‘three faces’ of power^(31)^. These dimensions of power are often mutually reinforcing – discursive power enables the development, deployment and maintenance of structural and instrumental power. Instrumental power involves wielding influence over others through direct action, fuelled, in part, by the use of resources^(32)^. Discursive power includes ‘controlling discourse, developing or challenging narratives and establishing new norms’^(31)^ whereas structural power is about defining the scope and institutional structures in which decisions are made (setting agendas and legitimising participation)^(31)^. Additionally, power is also conceptualised as embedded-in and operating through institutional arrangements, or the ‘rules of the game’^(33)^, both in visible and hidden ways^(34)^. It is of utmost importance that we seek to understand the power (im)balances between food systems stakeholders, the (dis)connections between formal and informal systems and the critical role of women, youth and marginalised groups in food systems. Increasingly, food is provided through an industrial food system that separates people from the source of their food and results in high rates of food insecurity, particularly for the most vulnerable in society. A lack of food and nutrition security is a symptom of a lack of power in a system that privileges free market principles over social justice and the protection of human rights.

Discourses encapsulate multiple perspectives that locate power more firmly in ideas, rather than people, systems or institutions and see power exercised through the ability to construct or control the framing or narratives around food insecurity and concerns regarding malnutrition in all its forms^(35)^. Notably, Foucauldian perspectives particularly emphasise the mutual embedding of power and knowledge in discourse^(36)^. Significantly, the concept of ‘discourse’ as underlying social action has been applied to the field of socioeconomic development and policymaking^(37)^; with scholars asserting that ‘studying discourses can reveal power relationships in society as expressed through language and practices’^(35)^.

To address the social dynamics more explicitly, we integrate concepts of social capital, which plays a critical role in the connection of people, the sharing of practices and the nature of mutual cooperation^(38)^. As such, the value of social capital can be assessed through the ‘networks, norms, and social trust that facilitate coordination and cooperation for mutual benefit [in the food system]’^(38)^. The most common distinction established when discussing social capital is between bridging, bonding and linking. Putnam^(39)^ suggests that bonding social capital is good for ‘getting by’ and bridging is crucial for ‘getting ahead’ whereas linking is characterised by relations between those within a hierarchy where there are differing levels of power^(39)^. Significantly, social capital can contribute to food security through the synergy that is created from the interrelationships among community members at every stage of the food supply chain from production to consumption. In fact, social capital is the benefits that society derives from the interaction between different networks and groups.

### Data collection

Semi-structured, in-depth interviews were conducted with forty-eight participants in order to collect data related to governance structures and power discourses influencing South Africa’s policy response to the multiple burdens of malnutrition. Each interview was conducted in English and lasted between 40 and 90 min, with key experts and professionals involved in South Africa’s food and nutrition policy sphere, in Cape Town, Johannesburg, Pretoria and East London (Table 2 – Appendix). Interviews were conducted between November 2018 and November 2019. Initial interviewees were identified through desktop research into key stakeholders and experts in the food system and further relied on snowball sampling. Participants were recruited through formal letters of invitation to individuals, heads of departments and organisations.

The semi-structured interview schedule consisted of a series of key, pre-identified topics with accompanying questions and prompts to allow for open discussions during dialogues, while ensuring all key research questions were covered. Questions included conceptions of the right to food by different actors; existing policy content and priorities across food system sectors and opportunities to strengthen action on nutrition (Appendix). The researchers conducted the interviews in-person, and all interviews were recorded using a digital audio recorder, with permission obtained at the beginning of each interview. Interviews were transcribed in full. Recruitment stopped once the researchers observed theoretical saturation. Theoretical saturation was reached when the complete range of constructs that make up our theory underpinnings were fully represented by the data. The data were iteratively analysed, which informed an assessment of theoretical saturation by the first and senior author. This study was approved by the Biomedical as well as the Humanities and Social Sciences Research Ethics Committee of the University of the Western Cape (BM18/7/20; HS19/5/33).

### Data analysis

After familiarisation with the raw data, interview transcripts were imported to NVIVO™ (version12·6, released in November 2019) and coded inductively and deductively by the first author. Deductively, once the codebook was developed and excerpts of data aligned with codes, we embarked on conceptual-driven coding applying our specific theoretical perspective. The codes and emergent themes were also reviewed iteratively during the coding process by two other authors. Codes included discourses on food system governance (nourishing, priorities and possibilities), the political economic context (social capital) and understanding spaces and forms of power (power types).

Data on code groups and quotations were exported onto Microsoft Word documents and documented in the results section that follows. Thematic analysis of the coded data was done with reference to the primary research question and the study framework. The first author led the analysis, in consultation with co-authors.

## Results

### Overview of findings

This analysis found that incoherent governance, power differentials and a lack of trust and social capital in food system governance were key emerging themes influencing effective nutrition governance in South Africa to address the multiple burden of malnutrition and fulfil the right to food. Important to note is that ‘power differentials’ and ‘social capital’ were predetermined codes.

#### The Problem: understandings of the right to food in the context of hunger and the double burden of malnutrition

All the interviewees indicated that the multiple contributors to poor food access and nutrition in South Africa make the right to food very difficult to operationalise. In particular, nutrition was not regarded as a right in itself despite the provisions of Section 28 of the constitution, but as an element of health or an outcome of a lack of access to food. Respondents highlighted a range of current policies implemented to address food access, including the school nutrition programme, social grants and nutrition-focussed policies such as taxation of unhealthy foods (health promotion levy (HPL)).

Many non-equivalent representations of food systems by different actors were evident in our analysis, leading to different assessments of both the nature of the policy challenge (in relation to all forms of malnutrition and the right to food), and the nature of appropriate policy solutions with respect to the food system. Conversations with interviewees revealed that very little data are available on how food systems work at different levels and what their outcomes are in the country. As such, only partial knowledge is available to help decision-makers influence the system and drive it towards more nutritious and food secure outcomes. In a complex context with multiple social, political and economic dimensions one interviewee stated: *…You’ve got justice issues, you’ve got power, you’ve got control, you’ve got a lack of resilience, too few players, big players, you have questions about policy uncertainty… – [Interview 43, Academia, Human Rights]*

### Power differentials influencing current governance structures

#### a) Spaces where relevant decisions are made and power is exercised

Interviewees from the health sector noted that the primary accountability lever for the government to hold the food industry to account is through legal mechanisms that would prevent recent dramatic episodes of food borne disease accidents and outbreaks (which have raised concerns about the effectiveness of current food control systems in protecting the consumers). It is this strength of the legal levers that explains why public health experts globally consistently call for a regulatory approach to improve the healthiness of food environments^(40)^, especially where existing deregulated conditions have created market failures, such as with stunting in children and obesity amongst adults in South Africa. There was clear recognition of the importance of nutrition, and the critical role of health policy, from the economic sector too. For example:

*I think the intent behind all government policy is actually very good. I believe that we can be measured up and compared with some of the best in the world, and I think particularly because we do take our lead from the international benchmarks… I believe that our Department of Health has the best interests of the public at heart. – [Interview 22, Private Sector, Trade]*

Notably, private sector interviewees indicated a preference for industry self-regulation by corporates and voluntary public–private partnerships, rather than mandatory regulations on the part of the government. This is a core aspect of laissez-faire neoliberal ‘governmentalities’ that most respondents found frustrating. Allowing corporations to set their own standards has so far translated into further regression of the right to food and nutrition. Alongside, even while introducing some initiatives designed to improve product quality like the HPL in South Africa or to regulate front-of-pack labelling, government policies continue to enable the food industry to market their products with exaggerated or essentially misleading nutrient and health claims. Many interviewees perceived that this resulted from a greater degree of access to decision makers by industry, compared to civil society. For example: *I don’t know whether it’s because of power relations or resources and so on… it’s the big food that has government’s ear, and civil society is virtually not there at all. – [Interview 17, Academia, Human Rights]*

Respondents from civil society pointed out that one way in which we can identify who is running the system is by looking at who is accumulating the greatest benefit from it. There is a small group of companies that regularly report not just profits, but growing profits. These profits, when contrasted against the losses of both farmers and consumers serve as a proxy for the power of the food industry in the governance of the South African food system. Respondents identified the need to consider the influence of corporations in structuring consumer perceptions on food quality and health. A few large corporations currently dominate the agricultural sector and the production, distribution, processing and marketing of food and its subsequent products. These companies were seen as being part of decision making processes. For example: *… the big manufacturers who are actually sitting in the room… they’re the people that pay to be part of these discussions, just to find out about regulation – [Interview 39, Private Sector, Public Health]*

Interestingly, it was unclear as to what the perceived normative value of the codification of the right to food was for those interviewees who were supportive of human rights. Right to food defenders located in activist spaces within civil society held the view that technically the right to food should be shaping government policy and expenditure, but there was no clear mechanism or understanding of how to operationalise it. For example:

*… the codification of the right to food at least provides a framework. It provides a reference point that you can use for an argument, for a motivation, to persuade people that this is not just your own opinion, or a bit of research that you did. It is something that is recognised by the highest level and authority of the government.” [Interview 27, Civil Society, Human Rights]*

#### b) Perceptions of who holds power and whose interests are represented

With respect to how power dynamics emerge in the food value chain and how they perpetuate food systems that favour dominant powerful actors, interviewees from academia and civil society believed that government is central to the food system transformation agenda since it holds the main lever of decision-making power (i.e. authoritative or institutional power). However, what also emerged from the interviews is a sense that the government is unlikely to act towards food systems transformation in the absence of visible support and pressure from other actors because the interests maintaining the status quo are too strong and government appears to lack capacity. The perspective of most of the participants was that powerful corporate players are actively shaping the availability, affordability and acceptability of foods and with this the broader consumer food environment. A respondent in civil society emphasised the following:

*It takes a form that we cannot see [corporate influence]… At the end of the day they make their decisions behind closed doors; when they publish the results they say this is confidential… – [Interview 19, Civil Society, Human Rights]*

Participants revealed that the vast majority of value captured within South Africa’s food system is by the retail sector, and this is contributing to poverty, inequality and food insecurity further up the value chain, particularly among producers. The overview of the food system here has to do with the structure that is the base of specific nodes of activity through which agro-food commodities pass and value is added, with a focus on corporate actors that operate across multiple commodity chains. Speaking to the fact that the largest node of activity is wholesale and retail, followed by food manufacturing and then primary agricultural production, interviewees within government pointed to an urgent need to examine who determines ‘value’ within the country’s food value chain. They shared the following on the situational analysis;

*Back in the days we used to have the marketing act or something that prescribed the marketing boards and these boards used to prescribe the prices of food commodities, and then since we have done away with that, we now have a problem. We have a serious problem in terms of food inflation, because those that are in the food industry seem to have the market concentration or market dominance… in the food market there are four big corporates that are dominating at the moment. I think they hold more than 87 % of the market share. – [Interview 29, Government, Agriculture]*

### Incoherent governance

#### a) Perspectives on the need for food system transformation as a governance challenge

Participants from academia and civil society identified the path to food system transformation as dependent on fundamental changes to governance, rights and power relationships, and many were frustrated at the absence of alternative discourses to current economic thinking dominating our food system. In contrast, respondents from business alluded to the viability of public–private partnership fixes. Food retail corporations were singled-out by participants in academia and civil society as having become key players in food governance because of their increasing economic power – a trend that implies that corporations have also acquired authority or legitimacy as political actors.

This is partly as a result of the unchallenged framing of the private sector and the market as beyond the government’s legitimate remit when it comes to food systems. Discursive power is expressed in the capacity of corporations to influence policies and political processes through shaping of norms and ideas. According to respondents located in academia these strategies are then further used to convey the efficiency and effectiveness of private institutions and standards for the benefit of the public good. For example:

*… They [corporates] pick up the food security language and the fear - and they promote that fear - how can we possibly feed that many people, because we [they] are the ones to do it … the ones that are doing it… And that’s the hegemonic discourse that has to be channelled into something else… So that’s what food security can lend itself to… they use it tactically for certain leverage. – [Interview 16, Academia, Human Rights]*

Civil society respondents also pointed out that food security is not just about producing food, but more about access to food and affordability, arguing that local governments in particular are compelled by the constitution to attend to food security challenges. The role of the district and municipalities should be largely to coordinate the efforts of key stakeholders such as the farming community and departments such as Agriculture, Health, Water and Sanitation and Social Development, among others, to work towards a common goal of ensuring food and nutrition security. The realisation of the right of access to food and nutrition is by no means a duty that is borne exclusively by national and provincial governments. The constitution allocates many functions to local government that offer points of leverage for municipalities to make meaningful contributions towards the creation of healthy food environments, but the government is yet to fully engage the sustainability of food systems within cities, communities and organisations in all their complexity – economic, ecological, political and cultural. Speaking to this, an interviewee in academia explained the following:

*We are misguided if we think policy is going to solve the question of the ills of the food system. I think it’s going to be a range of policies that entwine in different ways that ultimately enable local level responses where these systems cascade and collide with people, we’ve got to be moderating and working in those places; that’s why our cities are important and how do we enable cities… is it a food charter that we need, maybe? – [Interview 43, Academia, Human Rights]*

There is a gap in understanding how national commitments to nutrition are translated into sub-national implementation in South Africa. The split responsibility for food and nutrition policies between the ministries of Health and the Presidency has resulted in effective institutional homelessness for nutrition governance (i.e. with respect to food system policy) and the limited capacity and power of public health practitioners to influence the policy reform process. Running in tandem is the accountability challenge, resulting from the multiplicity of stakeholders and shared collective responsibility (or lack thereof) for results; and also, the messaging challenge, as multiple narratives are evident with respect to food system issues and solutions.

#### b) Perspectives on what needs to be done to improve food systems governance

All interviewees – despite having different articulations of the nature of the problem – identified that the current food system is not delivering acceptable food and nutrition outcomes, and that this represents an important challenge for South Africa. They also identified persistent challenges to achieving this as: the dominant paradigm, the changing nutritional context and the challenge of the shared governance that will be necessary to achieve improved nutrition. However, dialogue with interviewees revealed two schools of thought regarding the orientation of solutions to create healthy food environments and address the multiple burden of malnutrition in South Africa. In particular, civil society participants asked whether the responsibility of taking action lies with the individual (whilst the food industry offers more food choices) or with society (with the government providing societal leadership and advancing the right to food).

*… There’s a constant blame-shifting thing going on about who’s responsibility is it to implement and to figure out what’s going on, so I think there is a lot of segmentation in the nutrition scene in government, and that’s also one of the big problems why nothing gets done or decided-on. – [Interview 12, Civil Society, Public Health]*

Except for private sector respondents, a central narrative that was common amongst most participants was that the restructurings and reforms needed to redesign the food system towards more sustainability and regeneration are common with those that are needed to re-establish democratic responsibility in society. In this context, the role of the government as the principal duty-bearer of the right to food in the construction of food sovereignty was seen as critical. Government interviewees highlighted a few possibilities that could take place within our systems for the fulfilment of the right to food and nutrition through credible policy designs: better integration of smallholder farmers into the value chain, retail industry investment in nutrition and strengthening livelihood capacities.

*… The retail industry needs to do things differently… can we actually make nutrition a much more central focus… have a better range of produce at better prices and somebody might be willing to experiment with it and I think by doing that we can start changing the understanding and the boundaries of the way people think… but I think the intervention has to be thought through a logical, situational-specific context that’s tangible – [Interview 24, Government, Agriculture]*

A broad call for a rethink on the policy levers required for food system transformation was also made by a majority of respondents. In reference to attribution of responsibility for right to food violations most respondents in civil society and academia spoke of the need for an accountability framework to guide government and food industry engagement to address unhealthy food environments as part of a broader government-led strategy to address obesity and diet-related non-communicable diseases.

### Trust and social capital in food system governance

Our analysis of interview responses did not find any indication of robust social capital or social networks contributing to the processes, rules, practices and structures (both institutional and discursive) through which power and control are exercised and decisions are made. Instead, respondents in civil society placed emphasis on the need for social movements and grassroots engagement with governance arrangements that would ensure that their ‘voices’ are incorporated into decision-making.

*Food security requires good management and good governance and the ability to engage with the community… not the top-down things of you will do this and you will do that… I think that a lot of government problems are because they talk a lot but there is very little serious community engagement. – [Interview 27, Civil Society, Human Rights]*

We also found that different stakeholders for its action or inaction are increasingly holding the private sector accountable; most notable in this regard has been the work of the Competition Commission. However, while reputational damage may affect profit margins due to reduced sales, or production costs may rise due to fines and compensation payments linked to legal non-compliance, most social costs emanating from business malpractice remain hidden. Addressing private sector relations with the State in the realm of food and nutrition governance, respondents with knowledge of the State’s interface with business stressed the need for a more hands-on approach by government as it must engage all key stakeholders equally in the co-creation of a vision for a nourishing food system for the country.

In terms of the role of social capital in solutions, respondents pointed to the need for food systems and practices that are ethically grounded, scientifically verified, economically viable and clearly communicated. What was evident from the interviews is that government, as the principal duty-bearer for the right to food and nutrition, needs to acknowledge the context-specific nature of the social dynamics of food insecurity. The right to access sufficient nutritional food adheres to three intrinsic principles: the need to respect, protect and fulfil human rights. To achieve a food secure South Africa, participants from civil society suggested that government should lead legislative changes with the civil society sector acting as connectors between government and community members, the research sector could support food insecurity monitoring and evaluation while legal professionals should assist with the framing of human rights terminology and citizens should drive the political agenda, holding government to account. A government respondent’s comments further affirmed this line of thought *– “What I would like to see more, is us investing in building capabilities of people to be food secure with minimal help from the state… So building capacities, livelihoods of people. – [Interview 26, Government, Human Rights (Social Development)]*

Human rights defenders in academia and civil society emphasised the fact that malnutrition is political because it is multi-causal in nature and requires a multi-sectoral response because economic or technical solutions will not suffice. But the political dimension of the challenge of food systems transformation needs to be better understood and better addressed. Political will is needed to confront existing power relations to generate needed changes in production, consumption, waste disposal and other activities in the food value chain. One respondent stated: *It’s more about the political will to enact it. That’s the issue here… So the issue is not around a justifiable cause… It’s the political will to look at and change the current model in its framework. – [Interview 23, Civil Society, Human Rights]*

## Discussion

This analysis reveals that power in South Africa’s nutrition governance is concentrated among political and economic elites, who leverage neoliberal framing to minimise regulation and accountability. While the concept of nutrition governance is widely embraced, it remains poorly defined and inconsistently applied, often through narrow interpretations that hinder policy coherence and coordination. Consistent with previous research, we found that exclusionary policy processes reinforce the interests of powerful actors, particularly large food industry players, further entrenching inequality in the agro-food system^(20,35,41)^.

Although South Africa boasts strong legal institutions and social policies supporting socioeconomic rights, its minimalist regulation of the food value chain has allowed a small group of corporations to dominate agriculture and food production. This concentration of power, while often justified by cost efficiency, imposes significant social costs on poor consumers. Scholars have highlighted the inequities of the post-apartheid agro-food system, where elite economic decision-making has exacerbated disparities despite more inclusive formal political processes^(20,42)^. Aligning with this literature, our findings emphasise the need to embed the constitutional right to food within a more integrated, equity-focused policy approach to food and nutrition security.

The government’s reliance on piecemeal interventions has failed to address the structural issues driven by private sector dominance. Neoliberal policies often undermine stated health and nutrition goals, focusing instead on mitigating adverse effects rather than implementing systemic reforms. This echoes broader critiques of neoliberal food governance models in South Africa and other African contexts, where market-driven approaches deepen inequalities^(12,15,16,23,43)^. A reframing of ‘access to sufficient food’ as food justice, coupled with stronger advocacy and accountability efforts targeting both government and corporate actors, is urgently needed to ensure equitable access to nutritious food. In particular, politicising malnutrition will shift the conversation around food insecurity from a focus on agricultural production to looking at access to sufficient nutritious food. The Committee on World Food Security emphasises that the key is to shift the food system from a focus on production to a focus on consumption, empowering those marginalised in the system and supporting diverse distribution efforts^(44)^.

The challenge of addressing hunger and malnutrition requires innovative, systemic solutions that reduce reliance on fragmented, private-sector-led initiatives. Without stronger accountability and inclusion in food system governance, reforms risk perpetuating current inequalities. As suggested by the literature, an independent governance body modelled on Chapter 9 institutions could establish clear objectives, governance frameworks and performance standards to tackle the multiple burdens of malnutrition. Social capital, particularly trust-based collaboration, also emerges as a potential lever for addressing insecurity and hunger. Limited research has explored how social capital shapes lived experiences of hunger, but it could play a transformative role in fostering cooperative solutions and reducing the dominance of private sector actors in South Africa’s food system and more importantly, in ushering-in food democracy that would see a concerted effort to progressively realise the right to food for all South Africans^(45,46)^.

This study has also pointed to an opportunity for policymakers, activist-scholars and traditional academics to combat the lack of trust amongst the broad food system policy community in South Africa by actively creating ‘new’ deliberative food and nutrition governance spaces through embracing the ‘power to convene’. Convening is a powerful tool available to leaders who want to address complex problems that cannot be resolved without shared responsibility and joint action. At the time of writing, a Community of Practice fronted by the Centre of Excellence in Food Security meets regularly in the Western Cape and Gauteng provinces to discuss how to work together to bring change. Others are involved in the platform for multi-stakeholder dialogue convened at various spheres by the Southern Africa Food Lab^(47)^. Notably, the power to convene is not a new form of power. Rather, the concept enables right to food defenders and scholars to see governance opportunities and challenges in a new way^(32)^. ‘Convening takes place at the intersection of discursive and structural power, where it can be grounded in the power to reframe narratives through deliberation while enabling the construction of a new governance space’^(32)^. The power to convene is associated with Gaventa’s (2005) scholarship on claimed or created spaces, this is about resistance and solidarity with marginalised, non-state or non-market actors^(48)^. The realisation of the right to food and a coordinated response to the multiple burden of malnutrition in the country rests on the ‘power to convene’ – a process-oriented approach that increases activist-scholar’s capacity to mobilise; leverage different types of power; and integrate and coordinate, and build a systems-oriented vision by connecting across silos^(32)^.

The discussion effectively highlights the structural inequities and power dynamics shaping South Africa’s food and nutrition governance, offering a critical analysis supported by empirical evidence and relevant literature. It demonstrates a clear understanding of how neoliberal approaches and private sector dominance exacerbate inequality, while also proposing innovative solutions, such as reframing food access as food justice and leveraging social capital for systemic change. However, the discussion has limitations, including a lack of detailed exploration of how proposed interventions, such as an independent governance body or social capital initiatives, would be implemented in practice. Additionally, while the analysis draws on comparative insights from the literature, it could benefit from a deeper engagement with successful examples from other countries to provide more actionable pathways for reform. Lastly, while social capital is highlighted as a transformative tool, its potential remains underexplored, particularly regarding how trust can be rebuilt in a context of entrenched corporate dominance and a trust deficit in governance.

Without mechanisms to ensure accountability and equal participation, even convening processes risk being co-opted by powerful actors to maintain the status quo. Trust deficits between stakeholders – exacerbated by private sector dominance and historical inequalities – continue to hinder meaningful collaboration. As such, while the concepts of ‘social capital’ and ‘convening power’ are introduced as potential solutions, their reliance on trust and cooperative relationships may be difficult to achieve in this polarised context without strong, independent facilitation and clear governance frameworks.

### Conclusion

The codification of the right to food and nutrition in sections 27 and 28 of the South African Constitution is yet to be mobilised to create a credible normative and legal foundation for addressing the double burden of food insecurity and malnutrition. Inequity in access to food and in the distribution of the socio-economic benefits along the value chain is a major negative outcome of the current global food systems’ core activities^(49)^. At present, South Africa’s food system governance faces significant challenges, caught between powerful stakeholders maintaining the status quo and its obligations for realising the right to food and nutrition. Government intervention and leadership is necessary to address these obstacles and trans-disciplinary sectors must work collaboratively to ensure human rights strategies are incorporated into policy agendas to ensure progress is made to address malnutrition and hunger in the country and to contribute to the achievement of the United Nations’ Agenda 2030.

## Appendices

**Table 1.** Current and recent policies and initiatives relevant to food and nutrition in South Africa Table 1 long description.

Policy (title, year)	Lead department	Strategic objective	Other key departments and social partners
Current policies and initiatives			
The Southern Africa Development Community’s (SADC) Food and Nutrition Security Strategy, 2015–2025	Government of the Republic of South Africa (RSA)	The SADC Protocol on Health and the SADC Health Policy Framework 2000 commit to improving the nutritional status of the population in the region and addressing the SADC’s long-term goals of eliminating poverty. SADC member states have committed to meeting the nutrition targets in the Agenda for Sustainable Development by 2030 - in particular, Sustainable Development Goal (SDG) 2 (end hunger, achieve food security and improved nutrition and promote sustainable agriculture) and SDG 3 (ensure healthy lives and promote well-being for all at all ages).	All
National Development Plan: Vision 2030 (NDP), 2012	Office of the Deputy President & Department of Planning, Monitoring and Evaluation (DPME)	Identifies FNS as a key element of both poverty and inequality. As a result, the NDP makes reference to a number of steps that will improve food security, including the expanded use of irrigation, security of land tenure, especially for women and youth, and the promotion of nutrition education.	All
National Policy on Food Security and Nutrition for the Republic of South Africa (NPFSN), 2014	Office of the Deputy President & DPME	Seeks to establish a multi-sectoral Food and Nutrition Security (FNS) Council: to oversee alignment of policies, legislation and programmes, co-ordination and implementation of programmes and services that address FNS and draft new policies and legislation where appropriate.	DAFF, DSD, COGTA, DBE, DTI, DRDLR, StatsSA, provincial and local government, civil society and development partners
Household Food & Nutrition Security Strategy for SA, 2014 (The nature and the continued existence of this policy is unclear. It is uncertain by what means one can obtain a final official document of the policy. Interestingly, in 2014 the DSD circulated a draft discussion document for a Household Food and Nutrition Programme, which confusingly bore no resemblance to the Household Food and Nutrition Security Strategy. What happened to the proposed programme is unknown.)	Department of Social Development (DSD)	Aims to enhance production entitlements amongst subsistence producers; largely a subsidiary programme of the NPFSN^(49)^.	DPME, DAFF, DoH, local government, civil society
National School Nutrition Programme (NSNP), 1994	Department of Basic Education (DBE)	Aims to improve the health and nutritional status of the poorest learners in South Africa. Its main objective is to enhance learning by providing a daily nutritious meal at school. The programme is of great strategic importance; it involves a large financial commitment from government and reaches over 9 million learners.	DBE, NT, DSD, DPME, StatsSA, civil society communities
Fetsa Tlala Food Production Initiative, 2013	Department of Agriculture, Forestry and Fisheries (DAFF)	Seeks to promote food security and address structural causes of food insecurity, which continue to perpetuate inequality and social exclusion.	DRDLR, DWS, DTI, DSBD, NT, civil society and development partners
Social Relief of Distress Grant (food parcels), 2013	DSD	Provides ‘temporary assistance’ through the provision of food parcels or food vouchers to distressed households for a period of three months, with the possibility of extending it for a further three months. Applications for grants are processed immediately upon application and successful applicants receive either the food parcel or voucher on the spot.	NT, DPME, DoH, local government, development partners
National Environmental Health Policy, 2013	Dept of Health (DoH)	Aims to identify development needs in environmental health, particularly for populations that lack awareness and services due to historical imbalances, by outlining environmental health services. Promotes intersectoral collaboration in the provision of environmental health services by integrating environmental considerations with the social, political and development needs and rights of all individuals, communities and sectors.	COGTA, DEA, DAFF, DTI, NT, DSD, local government, civil society
The Integrated Growth and Development Policy for Agriculture, Forestry and Fisheries (IGDP), 2012	Department of Agriculture, Forestry and Fisheries (DAFF)	Aims to transform and restructure the agriculture, forestry and fisheries sectors, historically dominated by a small number of large companies; and to ensure that constraints experienced in input supply, production and marketing are addressed cost-effectively and in a timely manner.	DRDLR, DWS, DTI, DSBD, NT, civil society and development partners
Past policies and initiatives			
Roadmap for Nutrition in South Africa, 2013–2017	DoH	Sought to direct nutrition-related activities in the health sector by focusing on five strategies: (1) advocacy and technical support to integrate nutrition into relevant sector strategies and programmes; (2) positioning nutrition strategically within the health sector at national and provincial levels; (3) delivering key nutrition interventions through appropriate action; (4) strengthening the human resources to deliver effective nutrition services and (5) strengthening the information base for effective nutrition services.	COGTA, DSD, DRDLR, DAFF, DTI, NT, development partners, civil society
Strategy for the Prevention and Control of Obesity in SA, 2015–2020	DoH	Aimed to reduce the prevalence of obesity by 10 % by 2020 – through reforming obesogenic environments and enablers, while enhancing opportunities for increased physical activity and healthy food options in every possible setting, including healthcare facilities, early childhood development centres, schools, workplaces and the community at large.	DBE, DSD, DHA, civil society
Strategic Plan for the Prevention and Control of NCDs, 2013–2017	DoH	Aimed to increase healthy eating habits in the population through accessible and affordable healthy foods. Included the development of an integrated communication plan to influence people across the life cycle to make informed food and nutrition decisions.	DAFF, DTI, NT, DBE, DSD, DHA, civil society, development partners

Abbreviations: COGTA (Dept. of Co-operative Governance and Traditional Affairs), DAFF (Dept. of Agriculture, Forestry and Fisheries), DBE (Dept. of Basic Education), DEA (Dept. of Environmental Affairs), DHA (Dept. of Home Affairs), (DoH (Dept. of Health), DPME (Dept. of Planning, Monitoring and Evaluation), DRDLR (Dept of Rural Development and Land Reform), DSBD (Dept. of Small Business Development), DSD (Dept. of Social Development), DTI (Dept. of Trade and Industry), DWS (Dept. of Water and Sanitation), NT (National Treasury); StatsSA (Statistics South Africa).

**Table 2.** Summary of interviewees Table 2 long description.

Jurisdiction (*n* 48)	Agencies (*n* 48)	Sector (*n* 48)
National level (*n* 30)	Government (*n* 21)	Agriculture (*n* 11)
Western Cape Province (*n* 5) Eastern Cape Province (*n* 10)	Academia/research (*n* 10)	Trade/economics (*n* 7)
City of Cape Town (*n* 3)	Private sector (*n* 4) (Private sector respondents were drawn from an industry association that represents Retail and Manufacturing member companies in South Africa and directly from two of the largest food corporations in the country.)	Public health (*n* 13)
	Farming (*n* 2)	
	Civil society (*n* 11)	Human rights (*n* 17)

## Table 1 Long description

The table lists current and recent policies and initiatives relevant to food and nutrition in South Africa. It has 13 rows and 4 columns. The columns are labeled Policy (title, year), Lead department, Strategic objective, and Other key departments and partners. Each row provides details on specific policies and initiatives, including the title and year, the leading department, the strategic objective, and other key departments and partners involved. The table includes policies such as The Southern Africa Development Community Agriculture and Nutrition Security Strategy, 2015-2025, National Development Plan: Vision 2030, 2012, National Policy on Food and Nutrition for the Republic of South Africa, 2014, and others. Each policy is described with its lead department, strategic objective, and other key departments and partners.

Navigate back to Table 1.

## Table 2 Long description

A table summarizing interviewees by jurisdiction, agencies, and sector. The table has 48 rows and 3 columns. The columns are labeled 'Jurisdiction (n 48)', 'Agencies (n 48)', and 'Sector (n 48)'. The rows are grouped by jurisdiction, agencies, and sector. Row 1: National level (n 30), Government (n 21), Agriculture (n 11). Row 2: Western Cape Province (n 5), Academia/research (n 10), Trade/economics (n 7). Row 3: Eastern Cape Province (n 10), , . Row 4: City of Cape Town (n 3), Private sector (n 4), Public health (n 13). Row 5: , Farming (n 2), . Row 6: , Civil society (n 11), Human rights (n 17).

Navigate back to Table 2.

## References

[1] Pereira L & Drimie S (2016) Governance arrangements for the future food system: addressing complexity in South Africa. Environment 58, 18–31.

[2] Misselhorn A & Hendriks SL (2017) A systematic review of sub-national food insecurity research in South Africa: missed opportunities for policy insights. PloS One 12, e0182399. 28829787 10.1371/journal.pone.0182399 PMC5567909

[3] Sanders D & Reynolds L (2017) Ending stunting: transforming the health system so children can thrive. In South African Child Gauge, pp. 68–76 [ L Jamieson , L Berry and L Lake , editors]. Cape Town: Children’s Institute, University of South Africa.

[4] Bridgman G , van der Berg S & Patel L (2020) Hunger in South Africa during 2020: Results from wave 2 of NIDS-CRAM. Working Papers 25/2020. South Africa: Department of Economics, University of Stellenbosch.

[5] Statistics South Africa (2019) *Towards Measuring the Extent of Food Security in South Africa: An Examination of Hunger and Food Adequacy*. Pretoria, South Africa; available at https://www.statssa.gov.za/publications/03-00-14/03-00-142017.pdf (accessed September 2024).

[6] McLachlan M & Thorne J (2009) Seeding Change: A Proposal for Renewal in the South African Food System. Development Planning Division Working Paper Series No.16. Midrand, South Africa: DBSA.

[7] Nojilana B , Bradshaw D , Pillay-van Wyk V et al. (2016) Persistent burden from non-communicable diseases in South Africa needs strong action. S Afr Med J 106, 436–437. 10.7196/SAMJ.2016.v106i5.10776 27138654

[8] Drimie S & Pereira L (2016) Advances in food security and sustainability in South Africa. In Advances in Food Security and Sustainability, vol. 1, pp. 1–31 [ D Barling , editor]. Burlington: Academic Press.

[9] Statistics South Africa (2017) South Africa Demographic and Health Survey 2016: Key Indicator Report. Pretoria, South Africa: Statistics South Africa.

[10] National Planning Commission (NPC) (2012) National Development Plan Chapter 6: An Integrated and Inclusive Rural Economy. Pretoria, South Africa: The Presidency.

[11] Elver H (2016) The challenges and developments of the right to food in the 21st century: Reflections of the united nations special rapporteur on the right to food. UCLA J Int’l L Foreign Aff 20, 1–43.

[12] Moyo BH & Thow AM (2020) Fulfilling the right to food for South Africa: justice, security, sovereignty and the politics of malnutrition. World Nutr 11, 112–152.

[13] Ayala A & Meier B (2017) A human rights approach to the health implications of food and nutrition insecurity. Public Health Rev 38, 1–22. 29450082 10.1186/s40985-017-0056-5 PMC5810069

[14] Government of the Republic of South Africa (1996) Constitution of the Republic of South Africa. www.gov.za/sites/default/files/images/a108-96.pdf (accessed February 2025).

[15] May J (2017) *Food Security and Nutrition: Impure, Complex and Wicked*. Cape Town: Centre of Excellence in Food Security.

[16] Boatemaa S , Drimie S & Pereira L (2018) Addressing food and nutrition security in South Africa: a review of policy responses since 2002. Afr J Agric Resour Econom 13, 264–279.

[17] McLaren D , Moyo B & Jeffery J (2015) The Right to Food in South Africa: An Analysis of the Content, Policy Effort, Resource Allocation and Enjoyment of the Constitutional Right to Food. Johannesburg: Studies in Poverty and Inequality Institute (SPII).

[18] Kroll F , Swart EC , Annan RA et al. (2019) Mapping obesogenic food environments in South Africa and Ghana: correlations and contradictions. Sustainability 11, 3924.

[19] Thow AM , Greenberg S , Hara M et al. (2018) Improving policy coherence for food security and nutrition in South Africa: a qualitative policy analysis. Food Secur 10, 1105–1130.

[20] Greenberg S (2017) Corporate power in the agro-food system and the consumer food environment in South Africa. J Peasant Stud 44, 467–496.

[21] Ledger T (2016) An Empty Plate: Why We are Losing the Battle for Our Food System, Why it Matters, and How We Can Win it Back. Johannesburg: Jacana Media (Pty) Ltd.

[22] Swinburn B (2019) Power dynamics in 21st-century food systems. Nutrients 11, 2544. 31652523 10.3390/nu11102544 PMC6835350

[23] Durojaye E & Chilemba E (2018) Accountability and the Right to Food: A Comparative Study of India and South Africa. Food Security SA Working Paper Series No.003. South Africa: DST-NRF Centre of Excellence in Food Security.

[24] De Schutter O (2019) The political economy approach to food systems reform. IDS Bull 50, 13–26.

[25] Devereux S , Haysom G , Maluf RS et al. (2022) Challenging the Normalisation of Hunger in Highly Unequal Societies, IDS Working Paper 582. Brighton: Institute of Development Studies.

[26] Candel JJ (2014) Food security governance: a systematic literature review. Food Secur 6, 585–601.

[27] Claasen N , Van Der Hoeven M & Covic N (2016) *Food Environments, Health and Nutrition in South Africa Mapping the Research and Policy Terrain*, Working Paper 34 ed. Cape Town, South Africa: PLAAS, UWC and Centre for Excellence on Food Security.

[28] Pinstrup-Andersen P & Watson DD II (2011) *Food Policy for Developing Countries. Ithaca: Cornell University Press*.

[29] Anderson M , Nisbett N , Clément C et al. (2019) Introduction: valuing different perspectives on power in the food system. IDS Bull 50, 1–11.

[30] Hart TG , Davids YD , Rule S et al. (2022) The COVID-19 pandemic reveals an unprecedented rise in hunger: the South African Government was ill-prepared to meet the challenge. Sci Afr 16, e01169. 35340715 10.1016/j.sciaf.2022.e01169 PMC8938300

[31] Clapp J & Fuchs D (2009) Corporate Power and Global Food Governance: Lessons Learned. Corporate Power in Global Agrifood Governance, pp. 285–296. Cambridge, MA: MIT Press Journals.

[32] Clark JK , Lowitt K , Levkoe CZ et al. (2021) The power to convene: making sense of the power of food movement organizations in governance processes in the Global North. Agric Hum Values 38, 175–191. 10.1007/s10460-020-10146-1 PMC7458127 32904580

[33] North DC (1991) Institutions. J Econ Perspect 5, 97–112.

[34] Gaventa J (2006) Finding the spaces for change: a power analysis. IDS Bull 37, 23–33.

[35] Leach M , Nisbett N , Cabral L et al. (2020) Food politics and development. World Dev 134, 105024.

[36] Foucault M (2012) Discipline and Punish: The Birth of the Prison. New York: Knopf Doubleday Publishing Group.

[37] Mosse D (2005) Cultivating Development: An Ethnography of Aid Policy and Practice. London: Pluto Press.

[38] Brauer K (2010) Social capital, definition of. In International Encyclopedia of Civil Society, pp. 1388–1396 [ HK Anheier and S Toepler , editors]. New York, NY: Springer US.

[39] Putnam RD (2000) Bowling Alone: The Collapse and Revival of American Community. New York: Simon and Schuster.

[40] Gaventa J & Runciman C (2016) Untangling Economic and Political Inequality: The Case of South Africa, pp. 70. Paris: UNESCO Publishing.

[41] Ledger T (2016) Power and governance in agri-food systems: key issues for policymakers. Trade Ind Policy Strategies 23, 1–22.

[42] Moseley WG & Battersby J (2020) The vulnerability and resilience of African food systems, food security, and nutrition in the context of the COVID-19 pandemic. Afr Stud Rev 63, 449–461.

[43] Gaventa J (2005) Reflections on the Uses of the ‘Power Cube’ Approach for Analyzing the Spaces, Places and Dynamics of Civil Society Participation and Engagement. Participatory Methods. www.powercube.net/wp-content/uploads/2009/11/reflections_on_uses_powercube.pdf (accessed February 2025).

[44] May J (2020) Integrating a human rights approach to food security in national plans and budgets: the South African National Development Plan. In Exploring the Link between Poverty and Human Rights in Africa, pp. 15–29 [ E Durojaye and G Mirugi-Mukundi , editors]. Pretoria, South Africa: Pretoria University Law Press (PULP).

[45] Nosratabadi S , Khazami N , Abdallah MB et al. (2020) Social capital contributions to food security: a comprehensive literature review. Foods 9, 1650. 33198127 10.3390/foods9111650 PMC7698312

[46] Sodano V & Verneau F (2006) *Social Capital and the Food System: Some Evidences from Empirical Research*. ideas.repec.org/p/ags/eaae99/7764.html (accessed February 2025).

[47] South Africa Food Lab (2022) Southern Africa Food Lab. https://www.southernafricafoodlab.org (accessed September 2024).

[48] Welsh T (2020) ‘Radical Transformation’: COVID-19 Shows Urgency for Food Systems Policy Shifts. https://www.devex.com/news/radical-transformation-covid-19-shows-urgency-for-food-systems-policy-shifts-97579 (accessed September 2024).

[49] Department of Social Development & Department of Agriculture, Forestry and Fisheries (2014) *The National Policy on Food and Nutrition Security for the Republic of South Africa. South Africa*: Government of the Republic of South Africa.

